# Male and female anatomical homologies in the perineum of the dog (*Canis familiaris*)

**DOI:** 10.1002/vms3.128

**Published:** 2019-01-21

**Authors:** Margaret I. Hall, Jeffrey H. Plochocki, José R. Rodriguez‐Sosa

**Affiliations:** ^1^ Department of Anatomy College of Graduate Studies Midwestern University Glendale Arizona USA; ^2^ Department of Anatomy College of Veterinary Medicine Midwestern University Glendale Arizona USA; ^3^ Department of Medical Education University of Central Florida, College of Medicine Orlando Florida USA

**Keywords:** perineum, erectile tissue, penis, clitoris, external anal sphincter

## Abstract

Understanding the homologies between male and female perineal structure helps both evolutionary biologists and clinicians better understand the evolution and anatomy of canines. Domestic dogs (*Canis familiaris*) play an important role in human society, and canine perineal anatomy is important for maintaining dogs’ reproductive health for successful breeding and a wide variety of pathologies. Here, we investigate homologies between male and female perineal structure, identifying structures based on common function, anatomical relationships and attachments. In this investigation we dissected 21 male and female large‐breed dogs. We find broad structural homologies between male and female dogs related to erection, micturition and defecation, including muscles, fasciae and erectile tissue. Using these homologies will help anatomists and clinicians interpret the anatomical organization of the perineum, a notoriously difficult area of anatomy.

## Introduction

Domestic dogs, *Canis familiaris*, have important positions in society as companion animals, working animals and research models. A complete understanding of the reproductive health of dogs is mutually beneficial to the wellbeing of dogs and their owners, as well as being economically and socially important (Wells 2007; Friedmann & Son 2009; Hall *et al*. 2016). Accurate and complete descriptions of the dog perineum, the anatomical region between the pubic symphysis and caudal vertebrae, are important for understanding the evolution of mammalian reproduction and evolutionary relationships among taxa, as well as for successful breeding and surgical interventions related to infection, perianal fistulas, continence, urethrostomy, ovariohysterectomy, vulvovaginal cancer anal sac pathologies, vaginal prolapse and certain approaches to perineal hernia correction (Ashdown 1968; Vasseur 1984; Bilbrey *et al*. 1990; Hill *et al*. 2000; Smeak 2000; Morrison 2002; Ralphs & Kramek 2003; Vnuk *et al*. 2009; Fossum 2013; Cinti *et al*. 2015; Hall *et al*. 2017).

The current anatomical literature approaches dog reproductive biology by emphasizing genital and gonadal structures due to their relative importance to breeding and there has been relatively little attention especially to female external genitalia, including the anatomical structure of the vulva and the erectile body contributions to the clitoris (de Lahunta & Habel 1986; Done *et al*. 2009; Dyce *et al*. 2009; Evans & de Lahunta 2013, 2017). We suggest an examination of homologies between female and male perineal structure can augment our understanding of this complex anatomical region. This investigation aims to provide practical and thorough anatomical descriptions of the dog perineum. We hypothesize there are broad structural homologies between males and females related to erection, micturition and defecation, including muscles, fasciae and erectile tissues. Because humans represent the mammal for which perineal anatomy is most completely described, we discuss our findings in terms of structural homologies and relationships using standard human anatomical definitions (Plochocki *et al*. 2016; Netter 2017).

## Material and methods

We dissected 11 female and 10 male adult domestic dogs of various breeds of similar body size, including pit bull mixes, Labrador mixes and sheepdog mixes. All individuals were obtained from the teaching specimens utilized by Midwestern University College of Veterinary Medicine, which were supplied by Carolina Biological (Burlington, NC, USA) and preserved in Carosafe, or Ranaco Corporation (Tucson, AZ, USA) or Sargeant's Wholesale Biologicals (Bakersfield, CA, USA) and preserved with formalin, 3.7% formaldehyde, 1.2% methanol and <2% propylene glycol and phenol. Specimens were preserved via intravenous injection through either the internal jugular vein or the femoral vein and then supplemented by spot injections of formalin to the abdomen to ensure consistent preservation throughout the body cavity.

Dissection began by making an incision in the midline of the croup that continued ventrally along the lateral margins of the perineum to the pubis. Skin was reflected towards the midline in order to expose subcutaneous musculature. From there, deeper muscle and fascial layers were exposed, progressing to the pelvic diaphragm to reveal the perineal somatic musculature as defined in Done *et al*. (2009). In females, the vulva was transected either longitudinally or transversely in order to study the vulvar wall, external urethral orifice, external vaginal orifice, erectile tissue and the fossa, glans and body of the clitoris. In males, the bulb and shaft of the penis was transected to observe the urethra, erectile bodies and os penis in cross section.

## Results

### Both sexes

**Figure 1:**
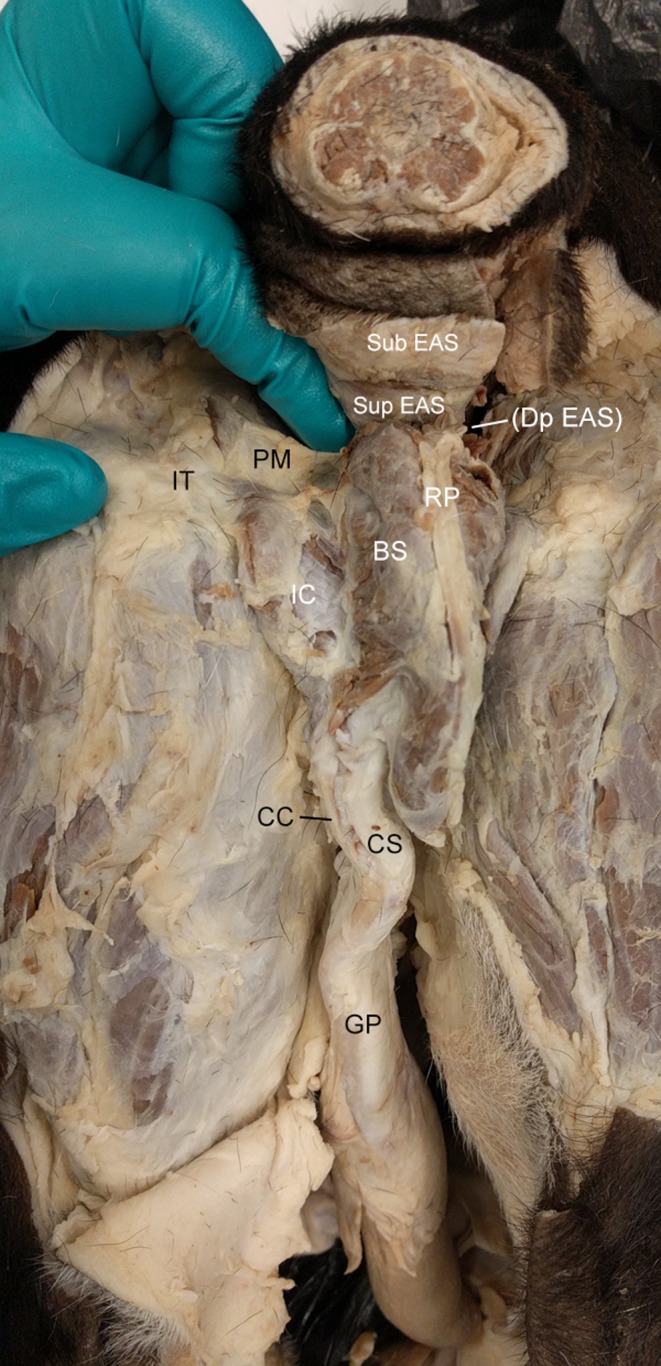
Caudal view of the male dog perineum. BS, bulbospongiosus m.; CC, corpus cavernosum; CS, corpus spongiosus; DP EAS, deep external anal sphincter m. (EAS); GP, glans penis; IC, ischiocavernosus m.; IT, ischial tuberosity; PM, perineal membrane; RP, retractor penis m.; Sub EAS, superficial EAS m.; Sup EAS, superficial EAS m.

The subcutaneous, superficial and deep portions of the external anal sphincter (EAS) form three distinct muscle layers, each with different fibre directions (Fig. 1). The relatively gracile subcutaneous EAS originates from, and inserts onto, the skin with narrow muscle fibres oriented from lateral to medial in a star shape surrounding the external anal orifice. The significantly more robust superficial EAS originates at the anococcygeal ligament to course ventrally on either side of the external anal orifice, and exhibits important sex‐specific continuities with the muscles associated with the genitalia (see below). Lateral to the superficial EAS we observed coccygeus exiting from its origin on the ischium and inserting onto the first caudal vertebrae. In all specimens, levator ani is divided into three distinct muscular bands: puborectalis, pubococcygeus and iliococcygeus. Deep to coccygeus, the deep EAS is continuous with the puborectalis portion of levator ani. A dense, thick connective tissue sheet, the perineal membrane, originates at, and spans the distance between, the ischial tuberosities just ventral to the superficial EAS and provides the point of origin for the erectile bodies of the phallus (Fig. 1). The perineal body, a connective tissue mass found between the anus and genitalia, was identified in all specimens caudal to the midline of the perineal membrane; however, no muscles insert on the perineal body, which occupies an anatomical plane caudal to the perineal muscles.

### Male

#### Muscles

The cutaneous trunci m. of the abdomen continues into the external lamina of the penile prepuce and attaches from lateral to medial around the prepuce orifice in a star shape, separate from, but similar in appearance and orientation to, the subcutaneous EAS. A prominent suspensory ligament extends from the ventral sheath of rectus abdominis m. into the prepuce of the penis that is associated with the glans penis. There is no direct attachment of the suspensory ligament to the body of the penis.

There are two discrete slips of the superficial EAS that extend into the genital portion of the perineum to form muscles associated with the phallic erectile bodies. A midline slip is continuous with the retractor penis m., which is formed by pennate fibres that attach to a tendon positioned superficially along the midline of the bulb of the penis. The medial portions of the right and left lateral muscular slips overlie the bulb of the penis and form the bulbospongiosus m., the two halves of which meet in a midline raphe deep to the retractor penis m. (Fig. 1). Some fibres from bulbospongiosus m. insert onto the tendon of retractor penis m. along its length. Ischiocavernosus m. takes origin from the ischial tuberosities and inserts along lateral side of the penile crura. Bulbospongiosus and ischiocavernosus mm. are continuous at the ischial tuberosities (Fig. 1).

#### Erectile tissues

**Figure 2:**
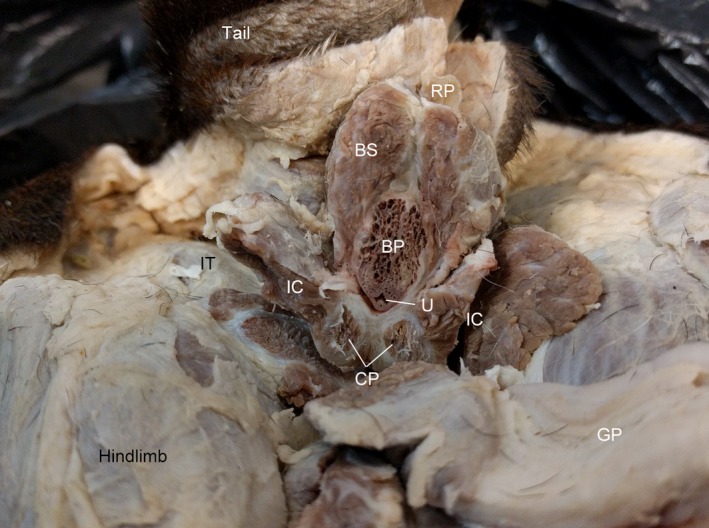
Caudal view of transected root of the penis of the male dog. BP, bulb of the penis; BS, bulbospongiosus m.; CP, crura of the penis comprised of corpora cavernosa and adjoined by the tunica albuginea; GP, glans penis; IC, ischiocavernosus m. (bisected on right side during dissection); IT, ischial tuberosity; RP, retractor penis m; U, urethra.

The erectile tissues originate at the dorsal margin of the perineal membrane and extend inferiorly to help form the body of the penis. The crura of the penis lie deep and medial to the ischiocavernosus m. on either side of the bulb of the penis (Fig. 2). Right and left crura remain discrete structures that are adjoined by a thick tunica albuginea. The bulb of the penis, which, at the level of the root of the penis, is dorsal and medial to the crura of the penis, contains the penile urethra and lies deep to the bulbospongiosus m. At the shaft of the penis, the crura are continuous with the corpora cavernosa on the dorsal aspect of the penis and the bulb of the penis is continuous with the more ventral corpus spongiosum. The os penis occupies the position of the corpora cavernosa in the glans of the penis. The corpora cavernosa do not extend into the distal glans penis.

### Female

#### Muscles

**Figure 3:**
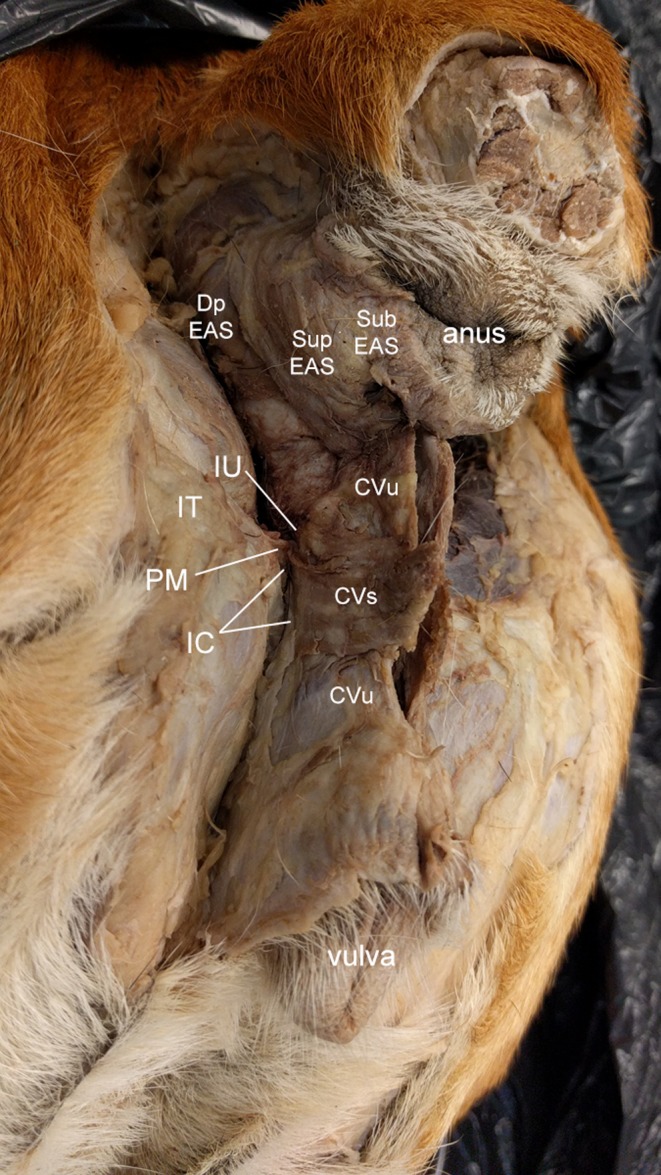
Caudolateral view of the female dog perineum. CVs, constrictor vestibuli m., CVu, constrictor vulvae m.; Dp EAS, deep external anal sphincter (EAS) m.; IC, ischiocavernosus; IT, ischial tuberosity; IU, ischiourethralis; PM, perineal membrane; Sub EAS, subcutaneous EAS m.; Sup EAS, superficial EAS m.

As in males, the superficial EAS originates from the anococcygeal ligament dorsal to the anus. Its fibres course ventrally and caudally in a robust band on either side of the anus and then continue to the genital portion of the perineum to create a continuous sheet of muscle, portions of which are associated with different genital structures (Fig. 3). The dorsal‐most fibres that originate as part of the superficial EAS meet at a midline raphe on the dorsal aspect of the vestibule, along with the more ventral muscle fibres, which continue along to insert on the vulva to create the constrictor vulvae m. Lateral fibres from the superficial EAS continue to the vicinity of the ischial tuberosities to join with the fibres that create the ischiourethralis m. We also observed the constrictor vestibulae m. originating from the ischial tuberosities, with parallel fibre direction, to course medially and dorsally to the dorsal aspect of the vestibule perpendicular to those of the constrictor vulvae m. and dorsal to the vulvar orifice and extending to cover about one‐third of the distance between the vulvar and anal orifices (Fig. 3). Constrictor vestibulae m. was consistently superficial to constrictor vulvae m., and the dorsal border of the constrictor vestibulae aligns with the dorsal aspect of the perineal membrane. Ischiourethralis m. and ischiocavernosus m. originate from the ischial tuberosities to extend caudally and ventrally onto the vestibule.

#### Erectile tissue

**Figure 4:**
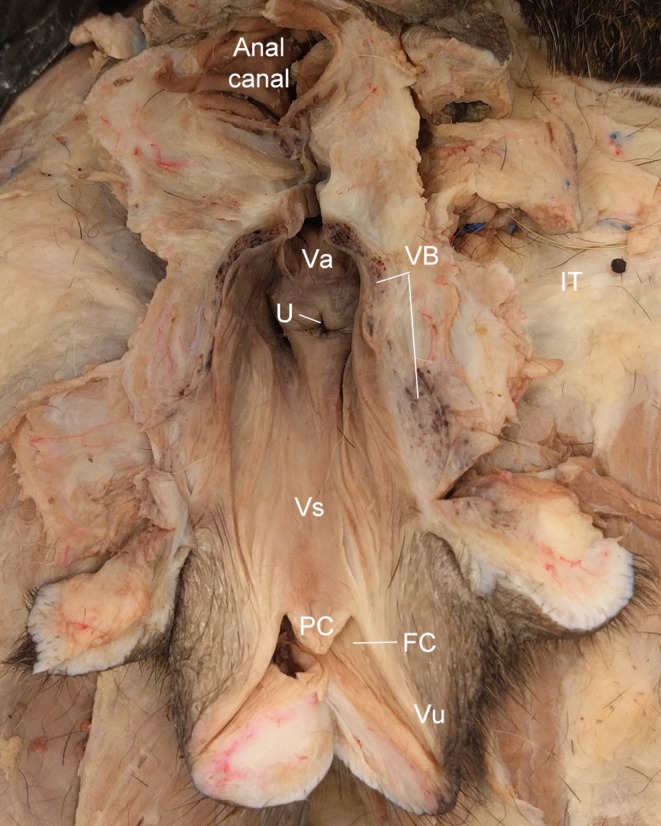
Dorsal view of a longitudinal bisection of the female dog vestibule and vulva. FC, fossa of the clitoris; PC, prepuce of the clitoris; U, external urethral orifice; Va, vagina; VB, vestibular bulb; Vs, vestibule; Vu, vulva.

**Figure 5:**
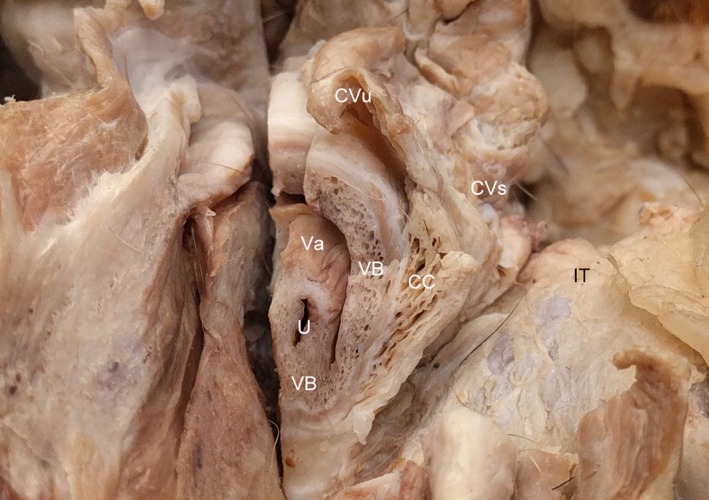
Transverse section of the vestibule at the level of the ischial tuberosity of the female dog. CC, corpus cavernosum; CVs, constrictor vestibulae m., CVu, constrictor vulvae m.; IT, ischial tuberosity; U, urethra; Va, vagina; VB, vestibular bulb. Note, the vestibular bulb supports the ventral urethra.

**Figure 6:**
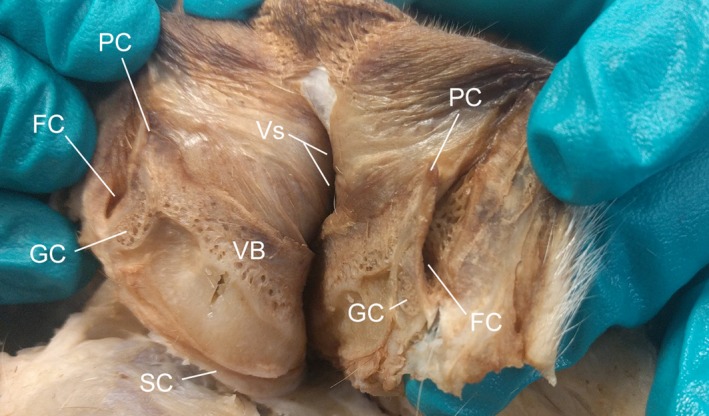
Midline sagittal section through the vulva of the female dog. FC, fossa of the clitoris; GC, glans clitoris; PC, prepuce of the clitoris; SC, suspensory ligament of the clitoris; VB, vestibular bulb; Vs, vestibule. Note (1) the continuity between the vestibular bulb and the glans clitoris, and (2) the prepuce contained within the fossa of the clitoris.

The vestibular bulbs originate at the dorsal aspect of the perineal membrane and then extend caudally to form the majority of the vestibular and vulvar walls (Fig. 4). The vestibular bulbs are bilateral at their origin, and then join in the midline to form a single erectile body that extends dorsally to partially support the ventral portion of the urethra, similar to the male (Fig. 5, see below), and then distally forms the glans clitoris (Fig. 6). There is another pair of erectile bodies, the roots or crura of the clitoris, that attach cranially on the perineal membrane and course dorsally along the lateral walls of the vestibular bulbs. Together, these erectile bodies, enveloped by dense connective tissue, form the substance of the vestibular and vulvar walls (Fig. 5). Distally, the two crura join to form the body of the clitoris, while the bulbs form the glans clitoris, which is overlain by an epithelial tissue covering, the prepuce of the clitoris, which extends into the vulva through the fossa of the clitoris (Fig. 6). A thickened band of connective tissue, the suspensory ligament of the clitoris, extends from the body of the clitoris in a sickle shape that arches ventrally and cranially to attach to the pubis (Fig. 4).

#### Vulva and vestibule

As described above, the vestibular walls are composed of erectile bodies overlain by a variably present fat pad (Fig. 4). When the clitoris is not erect, the prepuce of the glans clitoris in some specimens emerges slightly from the fossa of the clitoris, located in the ventral vulva approximately two centimeters cranially from the vulvar orifice (Figs. 4, 6). The external urethral orifice is located approximately two centimeters cranial to the fossa of the clitoris, and one centimeter caudal to the vaginal orifice (Fig. 4). The vagina is dorsal to the urethra (Fig. 4).

## Discussion

The purpose of this study is to augment the body of knowledge of male and female perineal anatomy in the adult dog. As hypothesized, our dissections reveal substantial homology between male and female perineal anatomy (Table 1). In both males and females, the EAS, rather than a single, compartmentalized, muscle as most commonly described, is composed of three separate muscles, a finding that has been observed in other mammals (Oh & Kark 1972; Shafik *et al*. 2007; Plochocki *et al*. 2016). Here, we refer to these muscles as (1) subcutaneous EAS, which acts primarily to regulate closure of the anal orifice, (2) superficial EAS, which also compresses the anal orifice, but is structurally and functionally continuous with bulbospongiosus in the urogenital portion of the perineum, and (3) deep EAS, which is continuous with levator ani. In dogs, levator ani is anatomically separated into three distinct bands. In humans, these bands are named for origin and insertion as puborectalis, pubococcygeus and iliococcygeus (Shafik 1975). Deep EAS is continuous with puborectalis, also similar to human structure (Bogduk 1996; Plochocki *et al*. 2016). Although muscle fibre orientation is different in dogs than in humans, the bony attachments are the same and therefore we suggest applying the same muscle names in dogs (Nitschke 1970). The separations between the EAS muscles allow for perineal herniation, especially in males, which lack the broad ligament of the uterus that may reduce hernia risk in females (Tobias 2015).

**Table 1:** Homologous perineal structures in the female and male adult dog

	Male	Females
Muscles	Bulbospongiosus	Constrictor vulvae
	Ischiocavernosus	Constrictor vestibulae, ischiocavernosus
	Ischiourethralis	Ischiourethralis
Erectile bodies	Corpus spongiosum	Vestibular bulbs, corpus spongiosum, glans of the clitoris
	Corpora cavernosa	Crura of the clitoris

Erectile tissues are also homologous between males and females. Embryologically, both males and females exhibit mesenchymal primordia from which four erectile bodies develop to create the structure of the male and female phallus (Kanagasuntheram & Anandaraja 1960; Moore *et al*. 2011). In males, bilateral corpora spongiosa fuse to create a single adult bulb of the penis that extends cranially to form the ventral penile shaft and the entire glans penis, both of which enclose the penile urethra. Bilateral corpora cavernosa remain discrete in the adult and form the dorsal aspect of the penile shaft. Proximally, the corpora cavernosa are adjoined by a tunica albuginea and is replaced by the os penis in the glans penis (Done *et al*. 2009). Here we find these erectile bodies take origin from a discrete fascial sheet, homologous to the human perineal membrane (Schimpf & Tulikangas 2005; Netter 2017). In female dogs, these same erectile bodies take origin from the perineal membrane, but instead of immediately forming a phallic shaft as in male dogs, the bulbs of the vestibule are configured to form the walls of the vulva, a new anatomical conceptualization that we describe here for the first time. In other words, instead of taking a bulbous form, the female corpora spongiosa are broad, sheet‐like structures that form the tube‐like structure of the vulva and vestibule, lined by mucosa and overlain with muscle (see below) and skin. Similar to males, in females we find bilateral corpora spongiosa combine to form a single vestibular bulb in the adult that extends to form a corpus spongiosum that creates the central portion of the vulva and glans clitoris. As in males, the bilateral corpora cavernosa lie lateral to the bulb; however, in females they reside within the vulvar wall, to which they are attached by a fascial layer similar in appearance to the tunica albuginea in males. Also similar to males, we find the female urethra supported ventrally by the corpus spongiosum, although this relationship is not maintained dorsally.

In both sexes the erectile tissues are associated with homologous muscles. Both males and females exhibit urogenital musculature that is continuous with the superficial EAS. In males, the superficial EAS contributes to retractor penis, bulbospongiosus and ischiocavernosus, all of which aid in the regulation of erection and micturition and, by continuations with superficial EAS, to defecation. In females, superficial EAS also contributes to the genital muscles, which current literature describes as retractor clitoris, constrictor vulvae, constrictor vestibuli and ischiocavernosus (Evans & de Lahunta 2013). Our dissection refines the definition of these muscles using homology with male structure based on sites of attachment and anatomical relationships with erectile tissues. The vestibular bulb, which forms the wall of the vulva, is overlain by a muscle defined as constrictor vulvae. We note here that it is homologous to the male bulbospongiosus. The retractor clitoris, as currently described (Evans & de Lahunta 2013), is part of the continuity between superficial EAS and constrictor vulvae. We observe that retractor clitoris and constrictor vulvae are a single, continuous muscle homologous to bulbospongiosus in males. Due to their continuities, they do not have separate functions, but work in unison to retract and constrict the vulva and apply pressure to the vestibular bulb to aid erection. This differs from Evans & de Lahunta (2013) who suggest constrictor vulvae and constrictor vestibuli are homologous with bulbospongiosus in males. Instead, we find that constrictor vestibuli and ischiocavernosus are a single muscle with a common origin to the ischial tuberosity and are together homologous to ischiocavernosus in males. Together, they serve as vestibular constrictors and retractors of the labia to help expose the clitoris during intromission and apply pressure to the corpora cavernosa to aid in female erection. In addition, these muscles help maintain intromission during ejaculation, which is unusually prolonged in canids relative to other mammals, as well as aid in male erection by constricting the penis to restrict venous drainage. Lastly, we suggest in both males and females, ischiourethralis is homologous to the superficial transverse perineal muscle in humans, as it arises from the ischial tuberosity and courses transversely between the urogenital and anal portions of the perineum and inserts onto the vestibule (Oh & Kark 1972; Standring 2015).

It is our hope that this contribution to the literature regarding the dog perineum will aid the veterinary and evolutionary understanding of the dog perineum. In particular, due to the morphological complexity of the vulva and associated structures, description of the female perineum has been incomplete. However, using the comparative approach utilized in this study, we find that male and female anatomy is very similar. Our hypothesis that male and female perineal anatomy is homologous is supported by our findings.

## Source of funding

This project was funded by Midwestern University intramural funds.

## Conflicts of interest

The authors report that no conflict of interest is present.

## Ethics statement

The authors confirm that the ethical policies of the journal, as noted on the journal's author guidelines page, have been adhered to. No ethical approval was required as this is an article that utilized only cadaveric teaching specimens.

## Contributions

All authors conceived of the project, performed the dissections, and participated in the analysis. MIH and JHP wrote the first draft of the manuscript, which was proofed and improved upon by JRSR.
